# Polyphenols in the Mediterranean Diet: From Dietary Sources to microRNA Modulation

**DOI:** 10.3390/antiox10020328

**Published:** 2021-02-23

**Authors:** Roberto Cannataro, Alessia Fazio, Chiara La Torre, Maria Cristina Caroleo, Erika Cione

**Affiliations:** 1 Department of Pharmacy, Health and Nutritional Sciences, Department of Excellence 2018-2022, University of Calabria, Edificio Polifunzionale, 87036 Rende (CS), Italy; r.cannataro@gmail.com (R.C.); alessia.fazio@unical.it (A.F.); latorre.chiara@libero.it (C.L.T.); erika.cione@unical.it (E.C.); 2 GalaScreen Laboratories, Department of Pharmacy, Health and Nutrition Sciences, University of Calabria, 87036 Rende (CS), Italy

**Keywords:** polyphenols, nutraceutical, microRNA, epigenomic

## Abstract

It is now well established that polyphenols are a class of natural substance that offers numerous health benefits; they are present in all plants in very different quantities and types. On the other hand, their bioavailability, and efficacy is are not always well proven. Therefore, this work aims to discuss some types of polyphenols belonging to Mediterranean foods. We chose six polyphenols—(1) Naringenin, (2) Apigenin, (3) Kaempferol, (4) Hesperidin, (5) Ellagic Acid and (6) Oleuropein—present in Mediterranean foods, describing dietary source and their chemistry, as well as their pharmacokinetic profile and their use as nutraceuticals/supplements, in addition to the relevant element of their capability in modulating microRNAs expression profile.

## 1. Introduction

Angel Keys, a biologist and physiologist who based his conclusions on his studies focusing on the dietary habits of people living in Southern Italy, was the first to present the phrase “Mediterranean diet” to the popular imagination, investing it with a scientific and cultural meaning. Now, following the joint candidacy of Italy, Spain, Greece and Morocco, followed by Cyprus, Croatia and Portugal, it has the recognition of not only UNESCO, but also of the WHO and FAO. The Mediterranean diet varies by country and region, so it has a range of definitions. In general, however, it is high in vegetables, fruits, legumes (such as beans), nuts, cereals, fish and unsaturated fats (such as olive oil). It usually includes a low intake of meat and dairy foods. In particular, some plants are characteristic of the Mediterranean vegetation; some have been present for thousands of years, such as olive trees, walnuts, oregano, pomegranates, onions and others including various citrus species [1]. The Mediterranean diet represents one of the first examples of a positive correlation between diet and cardiovascular health; in fact, a diet that involves the frequent eating of fruit and vegetables, possibly in season, in addition to seeds and olive oil, has been shown to exhibit significant benefits in health terms, resulting not only in preventing cardiovascular disease, but also diabetes, obesity and even various forms of cancer [2,3,4,5], although the dietary polyphenol intake in Europe seems to be high in the north [6]. This effect can be explained, at least in part, by the regular and varied intake of food polyphenols that characterize the Mediterranean diet. Although there is no set recommended daily dose, polyphenols have an important role in modulating and preventing various diseases, such as cardiovascular [6], as well as inflammatory diseases such as arthritis [7]. Probably the most important actions by polyphenols are carried out in the regulation and management of reactive oxygen species (ROS) and immunomodulation. The pathways that are influenced by much of them are those of nuclear factor kappa-light-chain-enhancer of activated B cells (NF-κB), mitogen-activated protein Kinase (MAPK) and arachidonic acids and phosphatidylinositide 3-kinases/protein kinase B (PI3K/AkT) as an inhibitor. On the other hand, they upregulate superoxide dismutase (SOD), catalase and glutathione peroxidase (GPx) expression: GPR40 [8,9,10,11]. 

Polyphenols are very often linked to the colors of the plants that contain them. They are present in practically all plant species and in various parts of the plant itself, especially in the leaves, fruits and roots [12]. On the other hand, while showing promising in vitro activity, they often present the obstacle of bioavailability, which does not always make them so useful if tested directly on humans. Polyphenols have a typical molecular structure with one or more aromatic rings, and one or more double bonds are present in the molecule. This structure guarantees an antioxidant action for all classes, as there is delocalization of the free radical itself, with consequent antioxidant activity. [12]. Together with this, polyphenols have a genomic and epigenomic action, in fact there are numerous studies that underline their regulatory action, among others, on NF-κB, MAPK and nuclear factor erythroid related factor 2 (Nrf2) [11,13,14]. In addition to that, they show epigenetic activity in modulating microRNAs expression and from this point of view the microRNAs could represent a useful evaluation tool to study polyphenols action in human. In this review, we choose six polyphenols—(1) Naringenin, (2) Apigenin, (3) Kaempferol, (4) Hesperidin, (5) Ellagic Acid and (6) Oleuropein—present in Mediterranean foods, as this dietetic lifestyle is linked to better health status [2,3,4,5].

## 2. Dietary Sources

### 2.1. Naringenin

**Figure 1:**
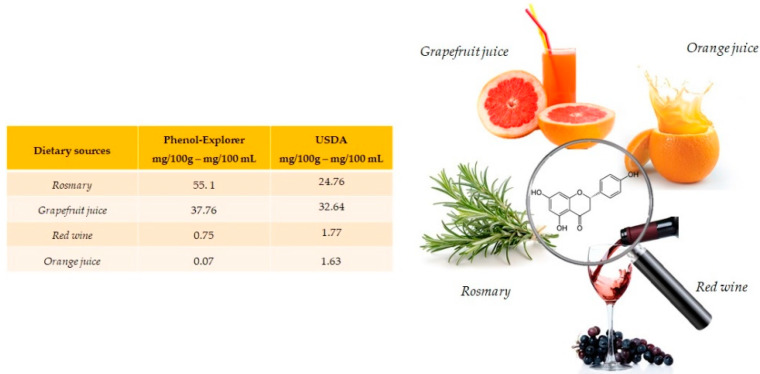
Main foods and beverages that contain naringenin, according to the database Phenol-Explore (http://phenol-explorer.eu/ accessed on 29 January 2021) and USDA Database for the flavonoid content of selected foods (https://www.ars.usda.gov/ accessed on 13 February 2021).

Naringenin is especially abundant in rosemary (55.1 mg/100 g) and present in grapefruit juice (37.76 mg/100 mL), red wine (0.75 mg/100 mL) and orange juice (0.07 mg/100 mL) (Figure 1). Naringenin is a flavonoid belonging to the subclass of flavanones, also often found in food in its glycosides form. [15].

### 2.2. Apigenin

**Figure 2:**
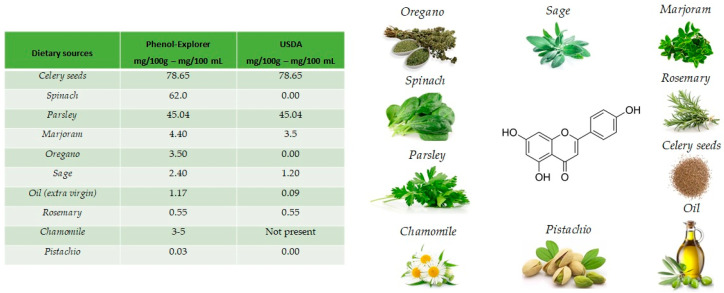
Main foods that contain apigenin, according to the database Phenol-Explore (http://phenol-explorer.eu/ accessed on 29 January 2021) and USDA Database for the flavonoid content of selected foods (https://www.ars.usda.gov/ accessed on 13 February 2021).

The name apigenin derives from the genus Apium in the Apiaceae also known as Umbelliferae and is found as a unique ingredient in chamomile (*Matricaria chamomilla*), an annual herb native to western Asia and Europe. Drinks prepared from chamomile contain from 0.8% to 1.2% of apigenin. Apigenin is abundant in a variety of other dietary sources [16], including fruits and vegetables (Figure 2), such as celery seeds (78.65 mg/100 g), spinach (62.0 mg/100 g), parsley (45.04 mg/100 g), marjoram (4.40 mg/100 g), Italian oregano (3.5 mg/100 g), sage (2.40 mg/100 g), chamomile (3 to 5 mg/100 g) and pistachio (0.03 mg/100 g), but the richest sources are the respective dried sources. [17].

### 2.3. Kaempferol

**Figure 3:**
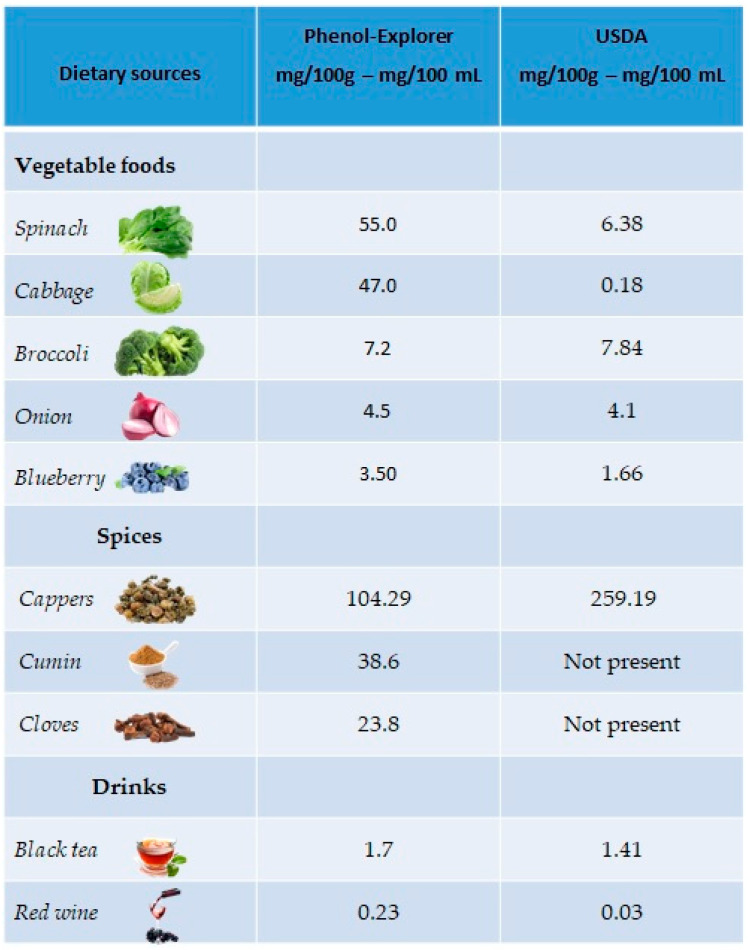
Main foods and beverages that contain kaempferol, according to the database Phenol-Explore (http://phenol-explorer.eu/ accessed on 29 January 2021) and USDA Database for the flavonoid content of selected foods (https://www.ars.usda.gov/ accessed on 13 February 2021).

The richest plant sources of kaempferol are green leafy vegetables, such as spinach (55 mg/100 g), cabbage (47 mg/100 g) and broccoli (7.2 mg/100 g), but also in onions (4.5 mg/100 g) and blueberries (3.17 mg/100 g). Regarding drinks, kaempferol is mainly present in black tea (1.7 mg/100 mL) and red wine (0.23 mg/100 mL). A good percentage is also present in spices such as capers (104.29 mg/100 g), cumin (38.6 mg/100 g) and cloves (23.8 mg/100 g) (Figure 3).

### 2.4. Hesperidin

**Figure 4:**
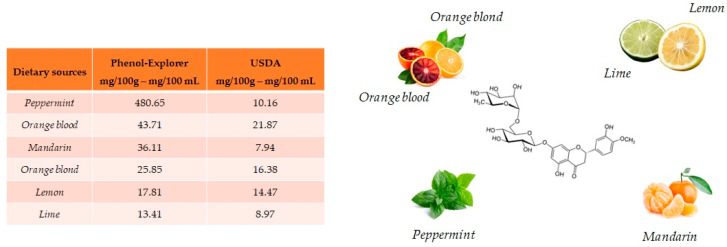
Main foods that contain hesperidin according to the database Phenol-Explore (http://phenol-explorer.eu/ accessed on 29 January 2021) and USDA Database for the flavonoid content of selected foods (https://www.ars.usda.gov/ accessed on 13 February 2021).

Hesperidin and its aglycone, hesperetin, are two flavonoids, which together with rutin and quercetin, are the main compounds of citrus fruits and, for this reason, this compound is called “citroflavonoid”. It is present mainly in blood orange (43.71 mg/100 mL), mandarin juice (36.11 mg/100 mL), blond orange juice (25.85 mg/100 mL), lemon (17.81 mg/100 mL) and lime (13.41 mg/100 g) (Figure 4). The presence of this compound has also been detected in plants; a high value is reported in peppermint (480.65 mg/100 g).

### 2.5. Ellagic Acid

**Figure 5:**
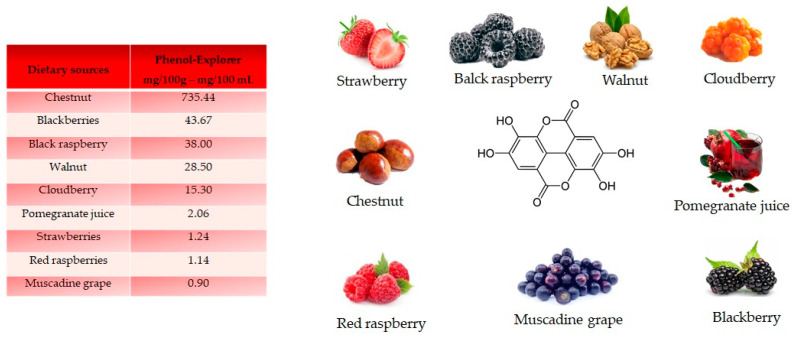
Structure and main foods that contain ellagic acid, according to the database Phenol-Explore (http://phenol-explorer.eu/ accessed on 29 January 2021). There is no value reported for the USDA Database for the flavonoid content of selected foods (https://www.ars.usda.gov/ accessed on 13 February 2021).

Ellagic acid, a dilactone of the dimer gallic acid, is a polyphenol found in fruit and vegetables. Some foods contain a more complex version called ellagitannin, which will be converted into ellagic acid by organism. Ellagic acid is prevalent in berries (Figure 5). Foods high in ellagic acid are chestnut (735.44 mg/100 g), blackberries (43.67 mg/100 g), black raspberries (38 mg/100 g), walnuts (28.50 mg/100 g), cloudberries (15.30 mg/100 g), pomegranate juice (2.06 mg/100 mL), strawberries (1.24 mg/100 g), red raspberries (1.14 mg/100 g) and muscadine grape (0.90 mg/100 g) [18]. Ellagic acid is synthetized by plants as a defense mechanism against infections and parasites.

### 2.6. Oleuropein

**Figure 6:**
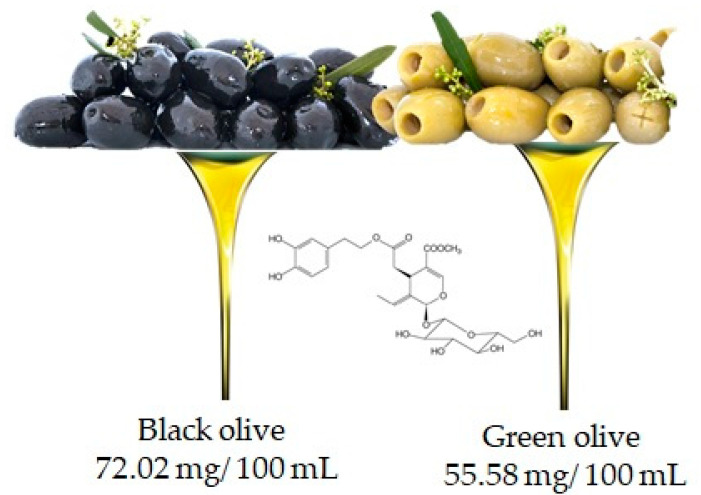
Olive is the main food that contains oleuropien, according to the database Phenol-Explore (http://phenol-explorer.eu/ accessed on 29 January 2021). There is no value reported for the USDA Database for the flavonoid content of selected foods (https://www.ars.usda.gov/ accessed on 13 February 2021).

Oleuropein is the molecule responsible for the bitter taste of olives and is the most common phenolic component in the leaves, seeds, pulp and skin of unripe olives. Although abundant, this compound undergoes hydrolysis during fruit ripening leading to the production of other important compounds such as hydroxytyrosol and ester derivatives [19]. It is important to underline that the oleuropein content may also depend on the variety of the olive (in fact black and green olives contain 72.02 mg/100 mL and 55.58 mg/100 mL, respectively (Figure 6)), but also, and above all, on the processing techniques used to obtain the oil [20].

## 3. Chemistry

**Figure 7:**
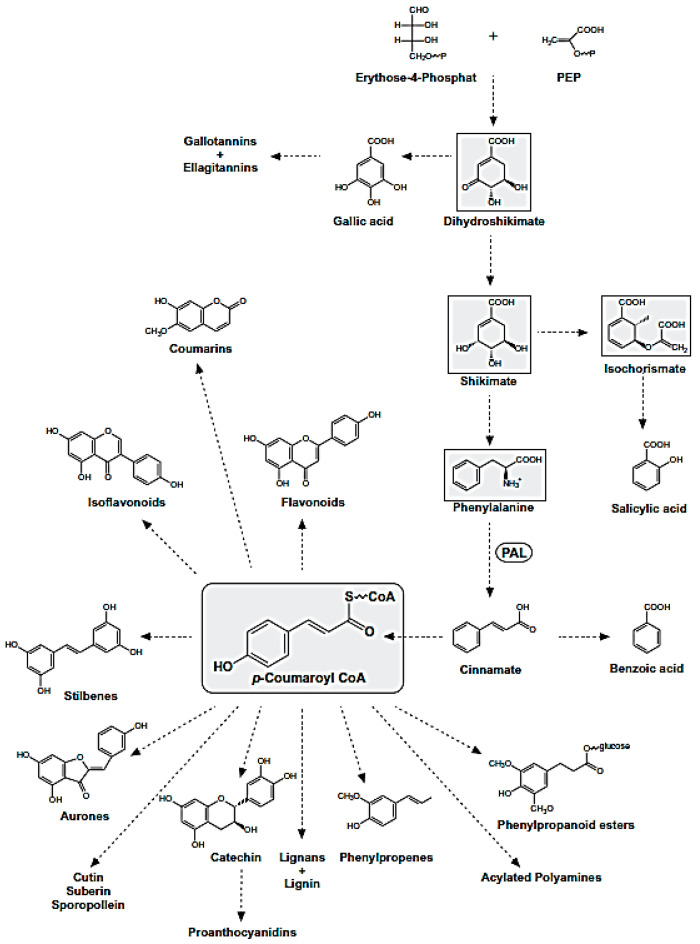
Phenylpropanoid Pathway. The metabolites of the shikimate pathway and p-coumaroyl CoA are shaded in grey.

Polyphenols are natural compounds synthesized exclusively by plants and characterized by two phenyl rings at least and one or more hydroxyl substituents. This description comprehends a large number of heterogeneous compounds with reference to their complexity. All phenolic compounds are synthesized through the phenylpropanoid pathway (Figure 7), starting from the amino acid phenylalanine [21], which is, in turn, a product of the shikimate pathway; the latter links carbohydrate metabolism to the biosynthesis of aromatic amino acids and secondary metabolites shikimate pathway [22].

The phenylpropanoid pathway leads to different classes of compounds which can be can be simply classified into flavonoids and nonflavonoids, or be subdivided in many subclasses depending on the structural diversity. This arises from the number of phenol units within the structure, substituent groups, variations in hydroxylation pattern and/or the linkage type between phenol units.

**Figure 8:**
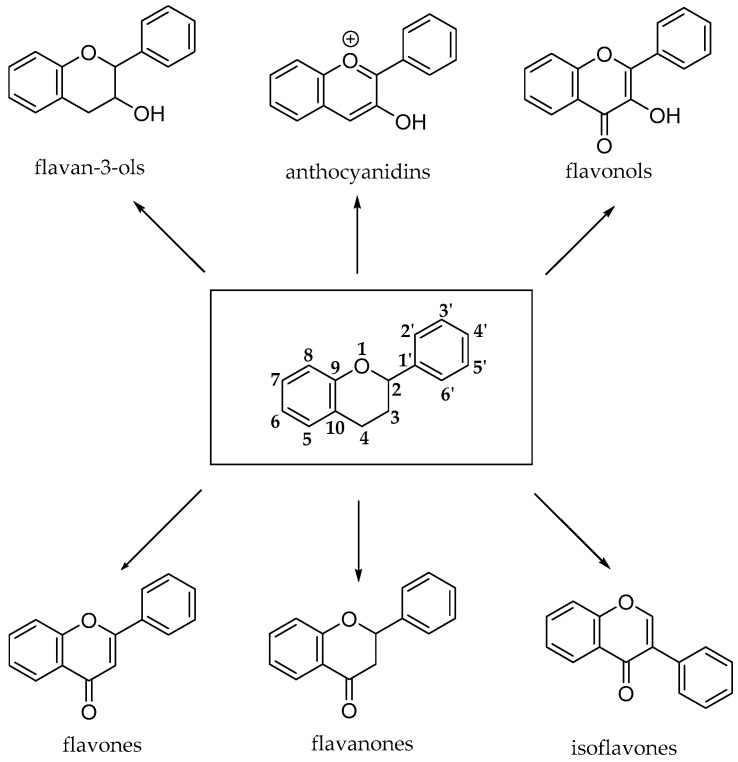
Basic structures of flavonoid subclasses.

The flavonoid pathway (calchone synthase) leads to the synthesis of six major classes of metabolites, such as flavonols, flavan-3-ols, anthocyanidins, proanthocyanidins and anthocyanins. All these compounds share the basic structure of diphenyl propanes (C6-C3-C6), in which phenolic rings (ring A and ring B) are usually linked by a heterocyclic ring (ring C), which usually is a closed pyran, as shown in Figure 8 [23].

Most flavonoids occur in edible plants and foods as β-glycosides, bound to one or more sugar units with the exception of flavan-3-ols (catechins and proanthocyanidins) and glycosylated isoflavones that are exposed to microbial β-glucosidases, which catalyze the hydrolysis of the glycosidic bond.

### 3.1. Naringenin

**Figure 9:**
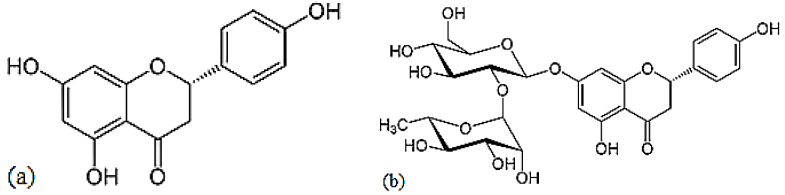
Structures of naringenin (**a**) and naringin (**b**).

Naringenin is a flavorless, colorless flavanone with three hydroxy groups at the 7, 5 and 4′ carbons (Figure 9) [24]. It may be found both in the aglycol form, naringenin (a), or in its glycosidic form, naringin (b), which has the addition of the disaccharide neohesperidose attached via a glycosidic linkage at carbon.

**Figure 10:**
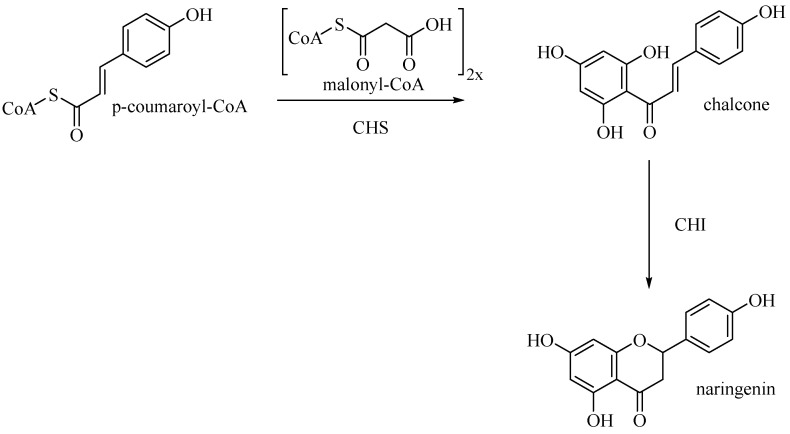
Biosynthetic pathway of naringenin.

Entering the flavone synthesis pathway, enzyme chalcone synthase (CHS) catalyzes consecutive condensations of three equivalents of malonyl CoA followed by aromatization to convert starting p-coumaroyl-CoA to chalcone [25]. Chalcone isomerase (CHI) performs stereospecific isomerization of tetrahydroxychalcone to (2S)-flavanone, which is the branch point precursor of many important downstream flavonoids, including flavones (Figure 10). 

### 3.2. Apigenin

**Figure 11:**
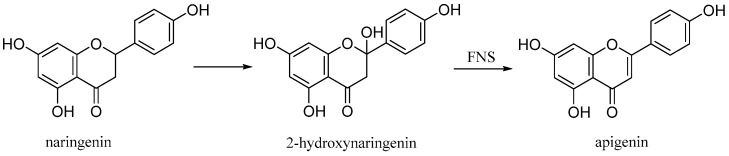
Biosynthetic pathway of apigenin.

Apigenin (4′,5,7-trihydroxyflavone), is a natural product belonging to the flavone class that is the aglycone of several naturally occurring glycosides. It is a yellow crystalline solid that has been used to dye wool. The approximately 650 known flavones arise from flavanones by oxidative processes catalyzed by a flavanone synthase (FNS) enzyme (Figure 11) [26]. Two types of FNS have previously been described; FNS I, a soluble enzyme that uses 2-oxogluturate, Fe2+ and ascorbate as cofactors and FNS II, a membrane bound, NADPH dependent cytochrome p450 monooxygenase [27].

### 3.3. Kaempferol

**Figure 12:**
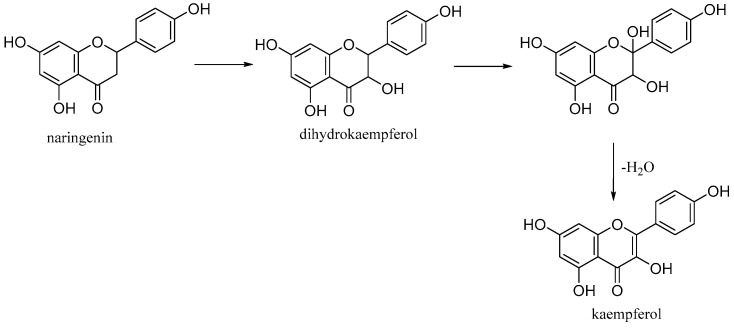
Biosynthesis of kaempferol.

Kaempferol (3,5,7-trihydroxy-2-(4-hydroxyphenyl)-4H-chromen-4-one) is a natural flavonol found in common foods derived from plants and fruits. It is biosynthesized from naringenin via a 2-hydroxy intermediate (Figure 12).

### 3.4. Hesperidin

**Figure 13:**
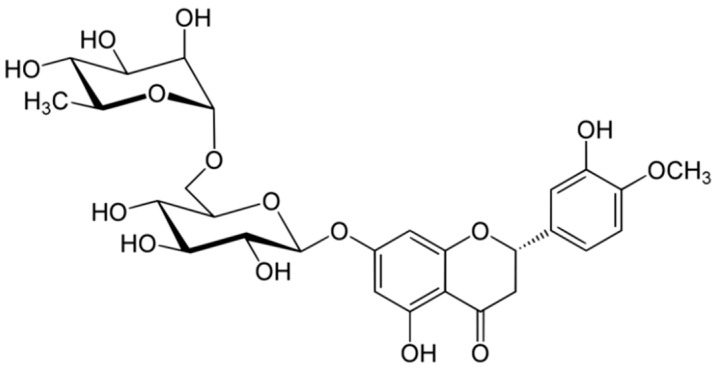
Structure of hesperidin.

Hesperidin is a flavanone glycoside found in citrus isolated in 1828 by French chemist Lebreton from the white inner layer of citrus peels (mesocarp, albedo) [28]. The structure consists of a flavanone aglycone, hesperetin, similar to naringenin, which differs from it for the different pattern of substitution on the B ring, which is functionalized with hydroxy group on 3′ carbon and methoxy group on 4′ carbon, whereas the naringenin lacks the methoxy group (Figure 13) [29].

### 3.5. Ellagic Acid

**Figure 14:**
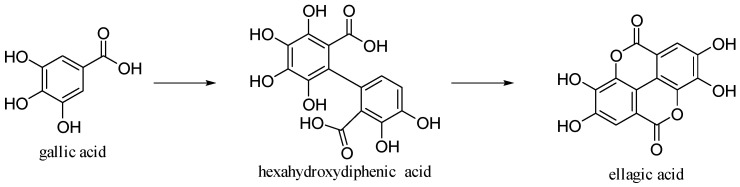
Gallic and ellagic acids.

Ellagic acid is 2,3,7,8-tetrahydroxy[l]benzo-pyrano-[5,4,3-cde] [1] benzopyran-5,10-dione [30]. It is formed by dimerization of gallic acid by oxidative coupling with intramolecular lactonization of both carboxylic acid groups; thus, it is a dilactone of the dimer of gallic acid (Figure 14).

### 3.6. Oleuropein

**Figure 15:**
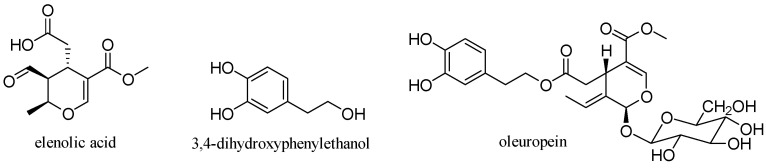
Structure of oleuropein.

Oleuropein is a glycosylated secoiridoid produced by secondary metabolism of plants and is present in all olive tissues. The term oleuropein is derived from the botanical name of the olive tree, *Olea europaea*. Oleuropein is an ester of elenolic acid with 3,4-dihydroxyphenylethanol (hydroxytyrosol), which is linked to a unit of glucose by a β-glycosidic bond (Figure 15) [31].

**Figure 16:**
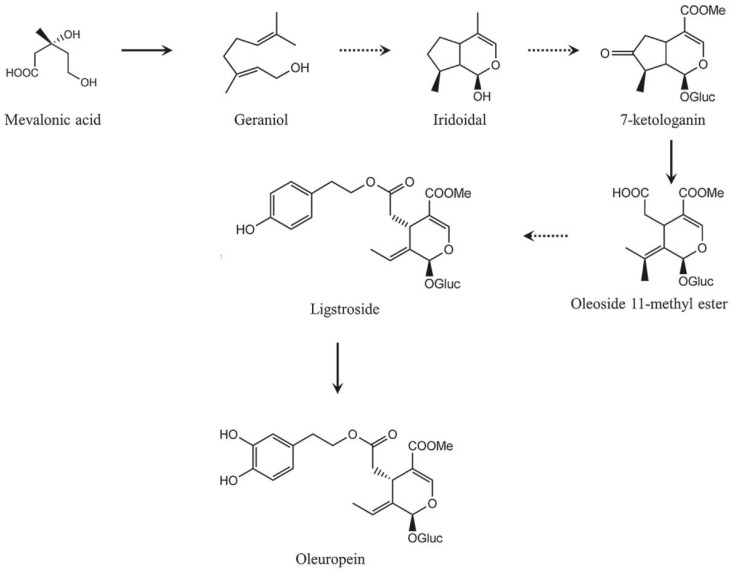
A simplified scheme of the oleuropein biosynthesis.

Secoiridoid conjugates that contain an esterified phenolic moiety, such as oleuropein, result from a branching in the mevalonic acid pathway in which terpene synthesis (oleoside moiety) and phenylpropanoid metabolism (phenolic moiety) merge [32]. This is illustrated schematically in Figure 16.

## 4. microRNAs

MicroRNAs (miRs) are a unique class of short endogenous RNAs. They are single-stranded non-coding RNAs able to modulate gene expression by binding to the complementary regions of 3′UTR sequence of specific mRNA targets. With this biochemistry, miRs allows mRNA degradation or inhibits translation. The peculiar regulatory capability makes them crucial for normal development in plants and animals [33]. In the area of molecular biology, it is generally accepted that miRNAs have evolved independently in distinct lineages. However, recent studies on miRs in non-bilaterian metazoans, plants and several algae raise the possibility that the last common ancestor of both lineages might have employed a miRs pathway for post-transcriptional regulation [33]. Antioxidant and microRNAs are an emerging field of research, especially in regard to polyphenols epigenetic ability [34]. To date, searching PubMed with the words “polyphenols and microRNAs” shows that 209 papers are currently present (accessed on 25 January 2021 at 7:00 p.m.). Focusing the search to “Naringenin and microRNAs”, “Apigenin and microRNAs”, “Kaempferol and microRNAs”, “Hesperidin and microRNAs”, “Ellagic Acid and microRNAs”, and “Oleuropein and microRNA” produced six papers for naringenin and microRNAs, 19 on apigenin and microRNAs, 22 on kaempferol and microRNAs, six on hesperidin and microRNAs, 14 on ellagic acid and microRNAs and eight on oleuropein and microRNAs. 

### 4.1. Naringenin

The polyphenols naringenin was tested in diverse cell systems displaying epigenetic property by regulating miRs, which in turn regulates the gene expression profile. 

Naringenin treatment protects trophoblasts and endothelial cells from the harmful high glucose environment by downregulating the miR-140-3p. In the interim, insulin receptor alpha and insulin-like growth factor 1 receptor expression were upregulated and the glucose uptake increased in naringenin treated trophoblasts and endothelial cells. Therefore, naringenin was proposed by Zhao et al. as a treatment candidate, for gestational diabetes [35]. Another evidence into the diabetes field by naringenin comes from its capability to ameliorated kidney injury through the let-7a. Yan et al. using mesangial cells (MMCs) and diabetic rats as experimental models demonstrate that naringenin led to an upregulation of let-7a under high glucose conditions affecting the expressions of fibronectin and collagen VI in MMCs. In addition, let-7a upregulation in renal tissue diminished the expression of transforming growth factor-β1 receptor 1 (TGFBR1), required for the regulation of Let-7a/TGFBR1 signaling pathway in diabetic nephropathy [36]. Naringenin has neuroprotective. In fact, in PC12 cells naringenin decreased the expression of miR-224-3p in a dose-dependent manner and increased the expressions of SOD1 mRNA and protein [37]. Furthermore, in rats, after spinal cord injury, the protective effect of naringenin was exerted through the repression of miR-223. This miR is a fine-tuner granulocyte production playing a fundamental role on neutrophils activation of the inflammatory response [38]. 

In lung cancer, naringenin biochemical activity inhibits migration and invasion, as well as tumor growth, through the regulation of the microRNA-3619-5p, which belong to a regulating axis with circular RNA FOXM1 and sperm-associated antigen 5 [39]. Lastly, Curti et al. recently demonstrate that naringenin is able to affect the miR-17-3p involved in the control of antioxidant endogenous system [40]. Using a Caco cell, a well characterized in vitro model which mimics the intestinal barrier, it is demonstrated that single enantiomers of naringenin R and S have similar activity on this miR, while their equimolar racemic mixture does not [41].

### 4.2. Apigenin

Apigenin in hepatocellular carcinoma cell line was noted to upregulate miR-520b and miR-101 to inhibit tumor growth. The miRs miR-520b and miR-101 are involved in Autophagy Related 7 protein (ATG7) and Nfr2 pathways, respectively, sensitize to doxorubicin treatment [42,43]. In neuron derivative tumor, miR 16, miR-138 and miR-423 were identified to be modulated by apigenin. In particular, miR-16 in glioma cells was upregulated negatively influencing the cell B cell CLL/lymphoma 2 (BCL2)/NFkB/Matrix Metallopeptidase 9 (MMP9) axis [44]. Similarly, in neuroblastoma cell, apigenin increased the expression of miR-138, while in glioma, stem cells knockdown miR-423 enhances sensitivity to apigenin involving the mitochondrial cell death pathways [45,46].

Apigenin could inhibit differentiation in TGF-β1-stimulated cardiac fibroblast, as well the synthesis of extra cellular matrix (ECM) component. This mechanism might partly be attributable to the reduction of miR-155-5p which control the c-Ski expression and the axis in which is involved, which might result in the inhibition of small mother against decapentaplegic (Smad)2/3 and p-Smad2/3 expressions [47]. 

Apigenin alleviates myocardial reperfusion injury (RI) in rats by downregulating miR-15b. This miR was found to be increased during myocardial RI, determining a downregulation of JAK2 that promotes myocardial apoptosis and ROS production, aggravating the myocardial injury. Apigenin treatment can downregulate miR-15b expression and increase the expression of Janus kinase 2 (JAK2) and the activity of Signal transducer and activator of transcription 3 (STAT3) pathway, reducing myocardial apoptosis and ROS production and alleviating myocardial RI [48]. Lastly, in mice, apigenin improved glucose intolerance by suppression of matured miR103 expression levels [49]. Apigenin normalized let-7f expression in epididymal fat tissues preventing colonic inflammation, associated with high fat diet-induced obesity [50]. Linked to lipids metabolism a liver-specific microRNA, miR-122, is also an important factor for hepatitis C virus (HCV), replication. Apigenin, in vitro, significantly reduced mature miR122 expression levels in a dose-dependent manner. Therefore, its intake, either via regular diet or supplements, may decrease HCV replication in chronically infected patients [51]. 

### 4.3. Kaempferol

In human lung cancer cells, kaempferol was able to up-regulate miR-340, along with the inactivation of the phosphatidylinositol-3-kinase (PI3K)/AKT pathways [52]. Likewise, in hepatocellular carcinoma cell line it remarkably reduces the expression of miR-21 leading to the inactivation of the same PI3K/AKT pathway [53]. Kaempferol is known to induce cardioprotective effects. In human aortic endothelial cells (HAECs), kaempferol induced the upregulation of miR-26a-5p, which, targeting the toll-like receptor 4 (TLR4), was able to inactivate the TLR4/nuclear factor kappa B (NF-κB) signaling pathway. This biochemical mechanism improves the oxidized low-density lipoprotein-stimulated HAECs [54]. In the same cell system, kaempferol increased miR-203 reversing the results led by lipopolysaccharides (LPS)-induced inflammatory injury [55]. In vascular smooth muscle cell (VSMC), kaempferol induces miR-21 expression inhibiting of cell migration [56]. The myocardial protective property of kaempferol was confirmed in ischemic heart disease (IHD), using primary cardiomyocytes and myoblast cell line H9c2. Under oxygen-glucose deprivation kaempferol exposure promoted the down-regulation of miR-15b, which target Bcl-2 and TLR4 [57]. Furthermore, kaempferol enhanced miR-21 level in H9c2 cells exposed to hypoxia/reoxygenation (H/R) and inhibition of miR-21 induced by transfection with miR-21 inhibitor significantly blocked the protection of kaempferol against H/R-induced H9c2 cell injury. Furthermore, kaempferol eliminated H/R-induced oxidative stress and inflammatory response by the decreases in ROS generation and malondialdehyde content, as well as the increase in antioxidant enzyme superoxide dismutase and glutathione peroxidase activities [58]. Kaempferol exerts anti-inflammatory activities and has been recognized as an effective agent for alleviating the clinical symptoms of osteoarthritis (OA) by decreasing miR-146a in the chondrogenic cell line ATDC5 activated PI3K/AKT/mTOR signaling pathway. In a rat model of OA, the expression of miR-146a in cartilage tissues was repressed by kaempferol [59].

In osteoblast precursor cell line MC3T3-E1, kaempferol enhanced the expression level of miR-101 promoting osteoblast proliferation, migration and differentiation [60].

### 4.4. Hesperidin

Aberrant oxidative stress was implicated in the environmental contaminant Di-(2-ethylhexyl) phthalate (DEHP)-induced testicular toxicity in which the miR-126-3p and the miR-181a are overexpressed. Hesperidin administration normalized their levels beside to other biochemical markers [61]. To the best of our knowledge, the present study demonstrated for the first time that the administration of hesperidin decreased the expression of ZEB2 by upregulating the expression of miR 132, which in turn promoted apoptosis and inhibited the proliferation of NSCLC cells [62]. Pre-treatment with hesperidin (25, 50, 100 mg/kg) for 7 days prevented these abnormalities induced by LPS injection. In contrast, this effect of hesperidin was abolished by a miRNA-132 antagomir. Taken together, these results suggest that the antidepressant-like mechanisms of hesperidin are at least partially related to decreased pro-inflammatory cytokine levels via the miRNA-132 pathway in the brain [63].

### 4.5. Ellagic Acid

In high glucose-induced T2DM HepG2 cells, ellagic Acid (EA) was able to elevate the miR-223 expression level, downregulating both, mRNA and protein levels of keap1. This led to the upregulation of Nrf2, SOD1 and SOD2 protein levels. Therefore, EA ameliorates oxidative stress and insulin resistance in the cell system used as model [64]. Another beneficial biochemical activity of EA was evident in ventricular remodeling after myocardial infarction. EA improved ventricular remodeling by up-regulating miR-140-3p expression and inhibiting MKK6 expression [65]. 

### 4.6. Oleuropein

In ovarian cancer was induced in xenograft mice model. Mice exposed to radiation with the simultaneous administration of oleuropein. Oleuropein sensitized ovarian cells to radiation altering the expression of miR-299. This miR was suppressed by a hypoxia inducible factor and relieved in response to oleuropein, which in turn suppressed heparanase 1 expression and consequently led to increased sensitivity to radiation due to synergistic effect of oleuropein with radiation [66]. Similarly, in nasopharyngeal carcinoma, oleuropein strongly enhanced radio-sensitivity through the downregulation of miR-519d [67].

## 5. Pharmacokinetic Profile

Results from a growing number of studies unveiled polyphenols as promising therapeutic agents due to their broad spectrum of biological activities; however, the effectiveness of these compounds in disease prevention and human health improvement is tightly related and limited to their bioavailability [68]. The concept of bioavailability encompasses several variables such as intestinal absorption, metabolism by gut microbiota, intestinal and liver metabolism, biological properties of metabolites, distribution at tissues level and excretion which in turn depend upon the chemical structure of xenobiotics. In addition, the various chemical forms of polyphenols lead to high variability in their rate and extent of intestinal absorption, as well as in the nature of circulating metabolites. Currently there is an increasing interest in biological properties of apigenin owing to it proved relatively low toxicity and effectiveness on cells with impaired biochemistry, such as cancer cells [69]. According to biopharmaceutics classification system (BCS) that correlates in vitro dissolution with in vivo bioavailability, Apigenin is categorized as BCS class II drug due to its low solubility and high permeability. Absorption of this compound occurs in the small intestine by both passive and carrier-mediated saturable mechanism [70]; furthermore, the gastrointestinal tract plays a crucial role in the metabolism and conjugation of apigenin before the entry of the compound into the systemic circulation and the liver [71]. It is worth mentioning that apigenin is naturally present in plants as glycosides. It has been hypothesized that apigenin glucosides can be hydrolyzed into apigenin by cytosolic β-glucosidase (CBG) and lactase-phlorizin hydrolase (LPH), which are enzymes produced by the liver, intestinal cells or the gut microbiota [70,72,73]. LPH has been shown to hydrolyze flavonoid glycosides and the resulting aglycone may then enter epithelial cells by passive diffusion [74]. The absorbed apigenin undergoes extensive Phase I and Phase II metabolism [75], accounting for the low bioavailability of the compound. 

### 5.1. Naringenin

The gut microbiota also plays a crucial role in the naringenin low availability as it determines extensive pre-systemic metabolism of the compound leading to the formation of degradation products such as phenolic acids [76]. As naringenin, hesperidin has limited bioavailability due to the presence of the rutosin moiety. The removal of either rutinoside or rhamnose from the molecule improves bioavailability and promotes a faster achievement of the maximum plasma concentration [77].

### 5.2. Apigenin

In both rats and humans, apigenin has been documented to produce glucuronide, sulfate conjugates or luteolin as major metabolites [78,79]. In addition, glucuronidation reactions also occur in the intestine and intestinal disposition may be more important than hepatic one in the first-pass metabolism of apigenin [78]. Of note, this natural flavone modulates efflux proteins, especially P-glycoprotein (P-gp) and metabolic enzyme CYP3A4, thus inducing clinically relevant drug-drug interactions by alteration of bioavailability and distribution profiles of targeted drug such as TK inhibitors or paclitaxel [72]. The elimination of Apigenin takes place through urine and feces and it is a slow process, therefore an accumulation of the flavone in tissues seems possible [72]. 

### 5.3. Kaempeferol

Kaempferol shows up a more favorable bioavailability profile. Absorption of the compound occurs in the small intestine through passive and facilitated diffusion or active transport [80]. Following absorption phase kaempferol undergoes metabolic transformation in the glucuronides and sulfoconjugates forms at both liver [81] and small intestine by enteric conjugation enzymes [80]. Kaempferol and its glycosides are also metabolized in the colon by the bacterial microflora that releases the aglycones and broke aglycone C3 ring to form compounds (i.e., 4-methylphenol, phloroglucinol and 4-hydroxyphenylacetic acid) that are either be absorbed and distributed to tissues by systemic circulation or be excreted in feces and urine [82,83,84,85]. Of note, combination of kaempferol with quercetin increase its bioavailability, thus improving its biological efficacy [86]. 

### 5.4. Hesperidin

Hesperidin bioavailability is also affected by various conditions including the health status [87] and the concomitant administration of this compound with other flavonoids such as quercetin, rutin, daidzein and chrysin [88]. Hesperidin requires deglycosylation into hesperitin by gut microflora to be absorbed. The absorption phase occurs at the level of colonocytes by proton-coupled active transport and transcellular passive diffusion [89,90]. Hesperitin is then selectively metabolized into the liver to eriodictyol by both the cytochrome P450 isoforms CYP1A and CYP1B1. Afterward, eriodictyol undergoes methylation and it is transformed to homoeriodictyol (3′-*O*-methylated) or hesperitin 4′-*O*-methylated. The other hesperitin metabolites comprise hesperitin glucuronides (7- *O*-glucuronide and 3′-*O*-glucuronide, hesperitin sulfates, 7-*O*- and 3′- *O*-sulfate, hesperitin sulfoglucuronides and homoeriodictyol glucuronides. It is worth mentioning that the first-pass metabolism of hesperitin occurs in intestinal cells leading to the formation of hesperitin 7-*O*-glucuronide and 3′-*O*-glucuronide, which represents the major hesperitin metabolites in vivo [91,92]. Hesperitin is also a selective inhibitor of cytochrome P450 CYP1B1 [93]. This observation provides a possible explanation of the compound anti-tumor activity being the enzyme involved in facilitating carcinogenesis process. Moreover, hesperitin enhances bioavailability of co-administered drugs such as diltiazem, verapamil and vincristine through inhibition of CYP3A4 or P-gp efflux [94,95,96]. The metabolites of hesperidin/hesperitin are detected in urine but not in feces, thus suggesting a further bacterial degradation to ring fission products and phenolic acids in the colon [97]. 

### 5.5. Ellagic Acid

The poor systemic bioavailability also affects the mechanism of action across conditions and doses of ellagic acid (EA) as demonstrated by several in vivo studies [98]. In fruits and nuts, EA exists in either its free form, as EA-glycosides, or bound as ellagitannin (ET) [99,100]. However, only a small portion of free EA is absorbed in the stomach, since ET are resistant to acidic pH. ET hydrolysis occurs in the small intestine, yielding to the release of EA. This latter is absorbed mainly by passive diffusion, although the involvement of a protein-mediated transport cannot be ruled out as suggested by in vitro experiment on Caco-2 cells line model [101]. In the systemic circulation, EA undergoes a massive first pass effect, being transformed in methyl esters, dimethyl esters or glucuronides, measurable in human plasma from 1 to 5 h after ET ingestion [102]. In the meantime, unabsorbed ET and EA fractions are mostly converted to a family of metabolites called urolithins by gut microbiota. Urolithins contain a common lipophilic moiety, thus resulting in a net improvement of bioavailability compared to EA [103]. However, the difference in gut microbiota composition leads to a wide variability in microbial metabolism of EA among individuals. Indeed, humans may produce no urolithins, highly active urolithins or less active urolithin, hence EA consumption may not exert equal health benefits in all subjects [104,105,106]. The low EA oral bioavailability was also confirmed in human pharmacokinetic studies demonstrating the rapid metabolism of the compound and the existence in the absorption phase of saturable mechanism [107]. 

### 5.6. Oleuropein

Poor data and often conflicting results exist on the oleuropein pharmacokinetic from EVOO or olive leaves in humans [108,109,110,111]. This discrepancy may be due to several factors. Indeed, the route of administration, genotype, age, sex, interaction with food and the different extraction processes deeply affect oleuropein bioavailability [112]. It has been reported that oral oleuropein ingestion is resistant to the stomach acidic pH and it is quickly absorbed in the small intestine, reaching a maximum plasma concentration earlier than conjugated metabolites of hydroxytyrosol in humans. Of note these latter represented the major fraction of the oleuropein phenolic metabolites in plasma and urine after intake [113], suggesting potential complete metabolization of oleuropein to hydroxytyrosol and other catabolic products. Attention has also been payed to the gut microbiota. In vitro and in vivo approaches demonstrated that oleuropein was rapidly deglycosylated to oleuropeinA by human fecal microbiota and then metabolized into elenolic acid and hydroxytyrosol by microbial esterase activity [114]. Further studies have shown that the conversion of oleuropein into hydroxytyrosol was performed by acid by Lactobacillus plantarum [115] and based on this observation oral granules for the co-delivery of Lactobacillus plantarum and a standardized olive leaf extract were developed in order to promote oleuropein metabolism and ensure high levels of hydroxytyrosol [116]. 

## 6. Health Effects 

### 6.1. Naringenin

Being present in foods known to be beneficial for health, such as citrus fruits and tomatoes, naringenin boasts numerous studies, even if, as happens for all molecules of plant origin, mainly in vivo and in vitro. In this sense, various studies show a favorable action in oncology, as it acts by limiting the progression of the cell cycle and angiogenesis; favoring apoptosis and acting directly on some carcinogens [117,118,119]. It also shows interesting qualities in the management of type 2 diabetes, obesity and metabolic syndrome; improving insulin sensitivity probably by regulating the action of AMPK, regulating the action of amylases and modulating inflammation via inhibition of NF-κB; beneficial actions are reported on the prevention of liver diseases in particular with an interesting mechanism, according to which naringenin should be incorporated in cell membranes thus providing a greater protective action [120,121]. Preventive action on neurodegenerative diseases can also be explained with the mechanisms proposed above [121]. Possessing immunomodulatory and antiviral activity, naringenin has also been proposed to support therapies against COVID-19 [122]. As for almost all polyphenols, the main problem is bioavailability, although many studies have used orange juice and not extracts or the molecule as it is; for this reason, different strategies are evaluated to make the molecule more bioavailable, as the doses that should be drawn from in vitro or in vivo studies should be between 25 and 50 mg per kg of body weight [123,124], even if some studies seem to confirm an absorption or at least a retention by the microbiota, with consequent beneficial action on it, with doses ranging from 200 to 500 mg [125,126,127]. In a case report by Murugesan et al., an improvement in insulin sensitivity is reported with an orange juice dosage of 150 mg naringenin for eight weeks [128]. In one of the few human studies 48 postmenopausal women took 210 mg of naringenin from grapefruit juice for 6 months, showing a clear benefit on arterial stiffness [129].

### 6.2. Apigenin

Like many substances of plant origin, apigenin has also been used for a long time through sources that are part of traditional medicines or common uses, remembering, in this regard, honey and chamomile [130,131]. There are many studies in which apigenin shows a very promising potential as an antioxidant [132] and as an adjuvant for numerous pathological states, such as diabetes, cancer, depression, amnesia and Alzheimer’s [131]; on the other hand, the studies on humans are few and the compound is extracted mainly from chamomile. Zick et al. tested a standardized chamomile extract that provided 15 mg of apigenin on 34 patients in double blind versus placebo, evaluating the quality of sleep and therefore the impact on insomnia, showing moderate positive effects: different quantities should probably be tested, as there are no adverse effects [133]. In two other papers [134,135], the effect of apigenin, still supplied as chamomile extract, on anxiety disorders was evaluated: the first followed a protocol with a variable dose from 8 to 13 mg of apigenin; in the second, a constant dose of 18mg of active ingredients, in both cases, showed positive effects, justifying their use in support of any drug therapy. A very interesting hypothesis comes from the work of Vollmer et al., from which it can be deduced that part of the apigenin is excreted intact with the feces; therefore, it is able to reach the microbiota present in the large intestine and thus positively influence the peculiar function [136].

### 6.3. Kaempeferol

Kaempferol is a polyphenol widespread in food, present in crucifers, but also in other foods used quite commonly in various food cultures. For this reason, there are also evaluations of epidemiological studies which positively correlate its intake with the prevention and treatment of various diseases [137]. A review Kashyap et al. [138] shows conflicting results from epidemiological studies: multiple positive results are highlighted, especially in the chemopreventive action, but not all studies report the same effects [139]. Human studies are not numerous, despite the promising results in vitro and in vivo [140,141,142,143]; epidemiological studies show a protective effect from cardiovascular diseases and a decrease in IL6 (with consequent modulation of inflammation) from a dosage of kaempferol included in a range of 2–12 mg per day, combined; however, with other polyphenols [139]; moreover, from an interesting study a protective effect on osteoporysis would result, according to various mechanisms that would lead to a decrease in adipogenesis, an increase in chondrogenesis and osteoblastogenesis [144].

### 6.4. Hesperidin

Hesperidin is the polyphenol most present in oranges and other citrus fruits, whose beneficial effect is well known, but often related to vitamin C and not to polyphenols. The beneficial effects demonstrated in vivo and in vitro are multiple, as for all polyphenols, thanks to their chemical structure, the antioxidant action is well present. In addition to this, a neuroprotective action was highlighted, beneficial on the cardiovascular system, bone health, on glycemic regulation and not least on the microbiota [145,146,147]; the dosages of hesperidin used in human studies vary from a minimum of 200 mg to a maximum of 500 mg, remaining, although largely in non-toxic or harmful dosages; some show improved cardiovascular health (64 subjects for 6 weeks 500 mg/day); however, positive effects are also shown in the management of hemorrhoids (70 subjects for 6 months of use), in neuroprotection (more than one study with dosages ranging from 200 to 500 mg/day), in the prevention and treatment of osteoporosis [148,149,150,151,152] and even in sports: 500 mg of hesperidin seems to improve aerobic performance in cyclists, probably thanks to the antioxidant action.

### 6.5. Ellagic Acid

This compound is very often linked to pomegranate; in fact, many papers refer to the use of the juice of the fruit, which, however, contains various polyphenols. In fact, in some cases the juice has proved more effective than the extract itself [153,154]. Common with all polyphenols, ellagic acid also has an antioxidant activity, directly and as a regulator of NF-κB [155,156]. In addition, in vitro and in vivo, it has also shown a regulatory action on inflammatory prostaglandins and on the onset of atherogenesis; many studies, however, are performed in vitro or in vivo. As for humans, a point to be clarified is the bioavailability and conversion of ellagic acid into urolithin, with relative efficacy of the latter [157,158]. It is interesting to note how the suboptimal absorption at the gastrointestinal level makes it available for the microbiota; from some papers, it seems that this favors some bacterial strains favorable for health, such as the Firmicutes: Bacteroidetes ratio [159]. The last study involved 20 subjects by administering 1000 mg of ellagic acid for four weeks. Another area where ellagic acid seems to have a positive effect is that relating to the central nervous system [160]; Ying et al. [161], administered to a group of 150 obese subjects, 50 mg of ellagic acid for 12 weeks, a positive effect was recorded not only on the lipid profile, but also on the BNDF, with improvement of the cognitive capacity of the subjects. Amma et al. [162], evaluated the action of ellagic acid in promoting recovery after an intense weightlifting workout; the study, carried out on nine subjects, showed a modulating effect on all the parameters relating to the antioxidant state, although it should be emphasized, however, that pomegranate juice was provided, and not ellagic acid alone. In the review of Huang et al., no positive effects of glycemic control are evident. 

### 6.6. Oleuropein

Oleuropein and its derivatives should represent the center of the Mediterranean diet, as a characteristic of the olive tree and consequently of the olive oil; the benefits, proven in vivo and in vitro are multiple and attributable to various pathways, to mention the antiatherogenic, cardioprotective, anticancer, neuroprotective, antidiabetic, anti-obesity, regulating lipids, antimicrobial and antiviral effects [163], but also others less frequently combined with polyphenols, such as the positive action on osteoarthritis [164] or inflammatory bowel diseases [165] and immunomodulation in general [166]; Somerville et al. [167], tested a supplement containing 100 mg of oleuropein on 32 high school athletes of good level, assessing their tendency to get sick and reported a positive correlation; De Bock et al. [168] demonstrated an improvement in insulin sensitivity by administering a supplement containing about 52 mg of oleuropein and 10mg of hydroxytyrosol to 46 obese subjects, in crossover versus placebo; with the same dosage, Lockyer et al. [169], on 16 subjects, analyzed the action of inflammatory cytokines and cardiovascular health, a modulating action was found in particular on IL8; overall, dosages of oleuropein from 50 to 150 mg per day and/or hydroxytorosol between 10 and 50 mg seem to be safe and effective to ensure an anti-inflammatory and antioxidant effect, which is beneficial in improving various conditions, such as atherosclerosis. Similar to other polyphenols [170,171]. 

## 7. Conclusions

Polyphenols certainly show great potential in assisting nutritionist or physician to co-treat various pathologies with a marked inflammatory component. Indeed, the numerous studies on the Mediterranean diet show how this eating style disfavors the onset of diseases that are the major cause of death worldwide, such as cardiovascular diseases, diabetes and cancer. The contemporary diet, in particular in the West, with the exception of north Europe, is often lacking of fruit and vegetables, the main sources of polyphenols; therefore, the consumption of these food categories should be strongly encouraged. On the other hand, the use of polyphenols as supplements could also have a strong impact on health. Although there are many in vitro and in vivo studies, those performed in humans are few and often not organized in a systematic way. Often the vegetable extract in *toto* or the vegetable is used, so it is difficult to understand which of the compounds had the predominant effect, also because they often act in synergy. In the current state of knowledge, we strongly recommend the use of fruit and vegetables in the diet and to consider the intake ranging from 50 to 200 mg/day of polyphenols, an amount that can also be doubled in pathological conditions, to ensure a beneficial action, at least from an anti-inflammatory and antioxidant viewpoint. As shown, polyphenols have an epigenetic action in particular on miRNAs, and this could also be a fascinating field of study to evaluate, in a clear and systematic way, even in clinical trials, the action of polyphenols.
